# Systematic review of transcriptome and microRNAome associations with gestational diabetes mellitus

**DOI:** 10.3389/fendo.2022.971354

**Published:** 2023-01-10

**Authors:** Kimberly A. Lewis, Lisa Chang, Julinna Cheung, Bradley E. Aouizerat, Laura L. Jelliffe-Pawlowski, Monica R. McLemore, Brian Piening, Larry Rand, Kelli K. Ryckman, Elena Flowers

**Affiliations:** ^1^ School of Nursing, Department of Physiological Nursing, University of California, San Francisco, San Francisco, CA, United States; ^2^ College of Biological Sciences, University of California at Davis, Davis, CA, United States; ^3^ College of Dentistry, New York University, New York, NY, United States; ^4^ Department of Epidemiology and Biostatistics, School of Medicine, University of California at San Francisco, San Francisco, CA, United States; ^5^ School of Nursing, Department of Family Health Care Nursing, University of California, San Francisco, San Francisco, CA, United States; ^6^ Earle A. Chiles Research Institute, Providence St Joseph Health, Portland, OR, United States; ^7^ Obstetrics and Gynecology, Reproductive Sciences, School of Medicine, University of California at San Francisco, San Francisco, CA, United States; ^8^ Department of Epidemiology, College of Public Health, University of Iowa, Iowa City, IA, United States

**Keywords:** mRNA, miRNA, transcriptome, miRNAome, microRNA (miR), gestational diabetes (GDM), differential gene expression (DGE), metabolic pathways

## Abstract

**Purpose:**

Gestational diabetes (GDM) is associated with increased risk for preterm birth and related complications for both the pregnant person and newborn. Changes in gene expression have the potential to characterize complex interactions between genetic and behavioral/environmental risk factors for GDM. Our goal was to summarize the state of the science about changes in gene expression and GDM.

**Design:**

The systematic review was conducted using the Preferred Reporting Items for Systematic Reviews and Meta-Analyses guidelines.

**Methods:**

PubMed articles about humans, in English, from any date were included if they described mRNA transcriptome or microRNA findings from blood samples in adults with GDM compared with adults without GDM.

**Results:**

Sixteen articles were found representing 1355 adults (n=674 with GDM, n=681 controls) from 12 countries. Three studies reported transcriptome results and thirteen reported microRNA findings. Identified pathways described various aspects of diabetes pathogenesis, including glucose and insulin signaling, regulation, and transport; natural killer cell mediated cytotoxicity; and fatty acid biosynthesis and metabolism. Studies described 135 unique miRNAs that were associated with GDM, of which eight (miR-16-5p, miR-17-5p, miR-20a-5p, miR-29a-3p, miR-195-5p, miR-222-3p, miR-210-3p, and miR-342-3p) were described in 2 or more studies. Findings suggest that miRNA levels vary based on the time in pregnancy when GDM develops, the time point at which they were measured, sex assigned at birth of the offspring, and both the pre-pregnancy and gestational body mass index of the pregnant person.

**Conclusions:**

The mRNA, miRNA, gene targets, and pathways identified in this review contribute to our understanding of GDM pathogenesis; however, further research is warranted to validate previous findings. In particular, longitudinal repeated-measures designs are needed that control for participant characteristics (e.g., weight), use standardized data collection methods and analysis tools, and are sufficiently powered to detect differences between subgroups. Findings may be used to improve early diagnosis, prevention, medication choice and/or clinical treatment of patients with GDM.

## 1 Introduction

Gestational diabetes (GDM) is associated with an increased risk for preterm birth and related complications for both the pregnant person and newborn (1). GDM is defined as chronic hyperglycemia that begins in the second or third trimester of pregnancy (1). The prevalence of GDM is increasing, and ranges from 1.7-11.6% internationally and from 2.5-7.6% in North America (2). GDM is often associated with pancreatic β-cell dysfunction that may lead to onset of diabetes after pregnancy (1, 3). Additionally, the offspring of pregnant people affected by GDM are more likely to develop attention deficit hyperactivity disorder, autism spectrum disorder, diabetes, and obesity (4–6). The pathogenesis of GDM is unclear, but evidence supports links between obesity, adipokines (the signaling molecules secreted by adipose tissue), or disruptions in oxidative stress mechanisms and GDM incidence (7, 8).

Changes in gene expression have the potential to characterize complex interactions between genetic and behavioral/environmental risk factors for GDM (8–10). Messenger ribonucleic acid (mRNA) are single stranded RNAs that translate genetic information into biologically active molecules. Given that mRNAs characterize, affect, and/or are associated with the expression of genes within an individual’s environment and context, they may be useful biomarkers of risk for GDM and potentially provide insights about the underlying mechanisms of risk (11). A previous review of the literature summarized genes that were differently expressed in GDM compared to other types of diabetes (8, 12). Differently expressed genes were influenced by GDM disease duration, obesity, number of gestations, glucose serum levels and the use of medications (12). However, prior studies have reported discrepant findings and it remains unclear how mRNA expression differs between blood samples from pregnant individuals with GDM relative to healthy individuals without GDM.

MicroRNAs, or miRNAs, are small, non-coding RNAs that interfere with mRNA translation to alter the expression levels of gene products (13, 14). Current biomarkers for GDM do not provide early detection of who is at greatest risk, do not characterize differences in the specific risk profile, and do not always predict resulting complications (15, 16). Circulating miRNAs are promising diagnostic biomarkers that represent environmental or behavioral influences on gene expression, potentially providing a more comprehensive measure of risk (15–19). A prior literature review summarized miRNA expression in the blood of pregnant people with GDM compared with healthy controls and found initial evidence that select miRNAs may be promising biomarkers for early detection of GDM risk, but concluded that there was insufficient overall evidence, particularly from samples of diverse individuals, to draw definitive conclusions (10).The purpose of this systematic review was to summarize the research about differences in circulating transcriptome (mRNA) and miRNA levels of adults with GDM compared with healthy controls. This paper updates previous literature reviews by incorporating both mRNA and miRNA studies in the synthesized analysis for a more robust understanding of the pathogenesis of GDM and associated complications.

## 2 Materials and methods

The systematic review was conducted using the Preferred Reporting Items for Systematic Reviews and Meta-Analyses (PRISMA) guidelines (19). The research question was formulated using the acronym PICOS, which stands for population, intervention/exposure, comparators, outcomes, and study designs. The study population is pregnant adults with GDM or adults with a history of GDM. The intervention/exposure in this case is an exposure to GDM during pregnancy. The comparator is adults without GDM. The study designs of interest were observational, cohort, quasi experimental, or experimental designs. The literature was searched by two independent reviewers (KL, JC) in PubMed for primary research articles about humans available in English from any date. The search terms, defined by a medical librarian experienced with systematic reviews, were as follows: “diabetes, gestational” [MeSH Terms] AND (“transcriptome” [MeSH Terms] OR “gene expression” [MeSH Terms] OR “micrornas” [MeSH Terms] OR “epigenomics”[MeSH Terms] or “Gene Expression Profiling”[Mesh]) AND ((humans[Filter]) AND (English[Filter])) AND ((humans[Filter]) AND (English[Filter])).

Included articles described the full transcriptome, the miRNAome, or a panel of at least three or more miRNAs in blood samples from adults with GDM compared with adults without GDM. Studies were excluded if they did not include a healthy control group for comparison, or if they were exclusively mechanistic studies conducted *in vitro* using a pregnant person’s blood samples.

Data were extracted about study and sample characteristics, study methods, instrumentation and data acquisition, validation, key findings, and limitations. This systematic review extracted the data based on the terminology reported by the authors of the studies included, including for race/ethnicity, sex/gender, and trimester in which the blood was drawn. A weighted mean and standard deviation were calculated using Excel software for the participant’s age, body mass index (BMI), and gestational age at the time of the blood draw, grouped by GDM or healthy controls. A formal risk of bias analysis was conducted using the Cochrane criteria for observational studies (20, 21).

## 3 Results

**Figure 1:**
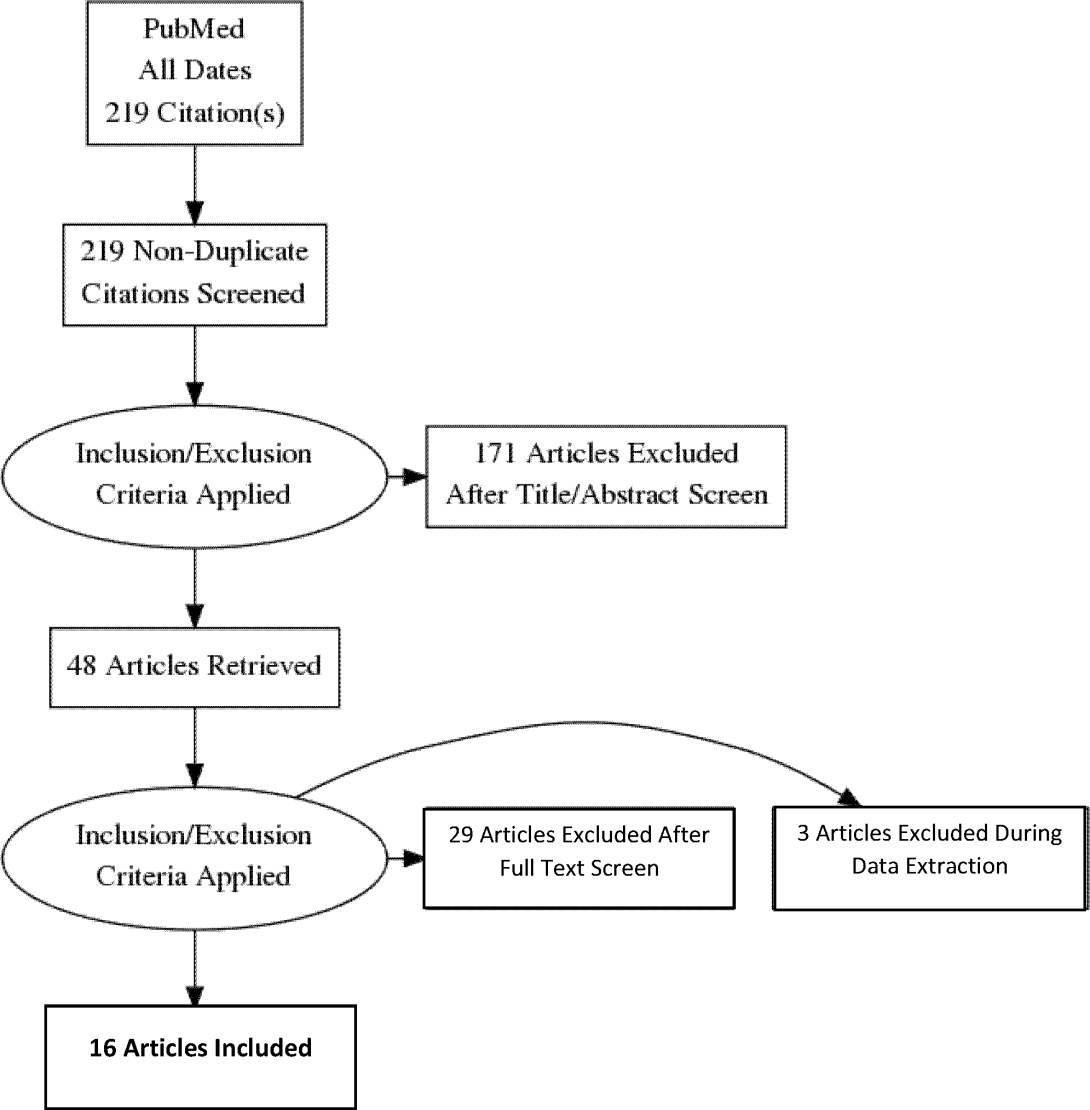
Preferred reporting Items for systematic reviews and meta-analyses flow diagram of the search strategy.

The PRISMA flow diagram provides an overview of the search strategy (**Figure 1**). After eliminating duplicates and applying inclusion and exclusion criteria, 16 articles were found representing 1375 adults (n=684 with GDM, n=691 controls) from 12 countries. Three studies reported transcriptome results and 13 reported miRNA-ome findings. An overview of the included studies and their key findings are presented in **Tables 1**–**4**.

**Table 1:** Summary of transcriptome studies included in the final sample.

Author (Year)	Study Purpose, Design, Setting	Sample	Instrumentation/Methods	Key Findings
Pappa et al. (2013)	**Purpose:** To investigate expression patterns of 10 major clock-related genes in the peripheral blood leucocytes of GDM women **Design:** Observational **Setting:** NR **Location:** Athens, Greece	**Sample:** *N* = 64 n = 40 GDM (20 GDM-Insulin; 20 GDM-Diet) n = 20 Controls n = 4 pregnant with T2D **Age (years)**: Range & Standard Deviation NR; 29.41 ± 4.07 (GDM-Insulin) 31.25 ± 4.42 (GDM-Diet) 29.09 ± 6.93 (Control) **BMI (kg/m^2):** Range & Standard Deviation NR; 22.77 ± 3.39 (GDM-Insulin) 21.83 ± 2.43 (GDM-Diet) 23.99 ± 4.34 (Control) **Gestational Age at Sampling (weeks):** NR, all blood draws done in 3^rd^ trimester **Race:** NR **Ethnicity:** NR **Nationality:** Greek, 100%	**Methods:** AllPrep DNA/RNA/Protein mini kit (Qiagen); RT-PCR mRNA transcript levels of 10 clock genes (*CLOCK1*, *BMAL1, PER1, PER2, PER3, PPARA, PPARD, PPARG, CRY1*, and *CRY2*) were compared **Validation**: NR **Tissue:** Peripheral leukocytes	No significant differences in mRNA levels between the subgroups of GDM-Insulin and GDM-Diet mRNA levels of *BMAL1, PER3, PPARD* and *CRY2* genes were significantly ↓ in both GDM subgroups compared to healthy controls *PER3* was significantly negatively correlated with HgbA1C Significant positive correlation between the expression of *BMAL1* versus *CRY2*, and between *BMAL1* versus *PPARD*
Steyn et al. (2019)	**Purpose:** To investigate potential GDM effect on gene regulation in fetal placental tissue and blood samples from mothers with GDM **Design:** Observational **Setting:** Chris Hani Baragwanath Academic Hospital **Location:** Soweto, South Africa	**Sample:** *N* = 12 n = 6 GDM n = 6 Control **Age (years)**: Range & Standard Deviation NR; 31.3 (GDM) 26.7 (Controls) **BMI (kg/m^2)**: Range & Standard Deviation NR; 37.9 (GDM) 30.8 (Controls) **Gestational Age at Sampling (weeks)**: Range 29-33; Mean & Standard Deviation by group NR **Race**: Black, 100% **Ethnicity**: NR **Nationality**: South African, 100%	**Methods:** Tempus™ Spin RNA Isolation Kit Protocol; RNA-Seq **Validation**: Q-RT-PCR, using *RPLPO, ACTB* and *HPRT1* housekeeping genes and 20 selected genes **Tissue:** Whole blood	1088 differentially expressed genes, reduced to 60 candidate genes; top gene ontology terms were binding activity and catalytic activity. Pathways most enriched were carbohydrate and NADP metabolism. Five genes (*G6PD, TKT, ALDOA, PGLS, DCXR*) clustered together with those pathways and code for enzymes in the pentose phosphate pathway. Negative correlations: * *G6PD* mRNA expression with maternal glucose at fasting, 1 hour, and 2 hours post-glucose load. *TKT* mRNA expression and maternal glucose levels at fasting and at 1-hour post-glucose load.*IGFBP-1* maternal glucose levels at fasting, 1- and 2 hours post-glucose load; and for *IGFBP-2* at fasting and 1-hour post-glucose load. There was a significant positive correlation of *IGFBP-1* mRNA expression in maternal blood with birthweight.
Zhao et al. (2011)*	**Purpose:** To determine what genes and/or pathways are associated with GDM in Chinese ethnicity patients **Design:** Observational **Setting:** Beijing Obstetrics and Gynecology Hospital **Location**: Beijing, China	**Sample**: *N* = 9 n = 5 GDM n = 4 Control **Age (years)**: Range 25-37; 30.0 ± 3.7 (GDM) 33.0 ± 3.6 (Control) **BMI (kg/m^2)**: Range: 23.4-31.9; 29.6 ± 2.1 (GDM) 26.0 ± 2.3 (Control) **Gestational Age at Sampling (weeks)** Range 23-41; 33.8 ± 7.3 (GDM) 37.5 ± 0.7 (Control) **Race**: NR **Ethnicity**: Chinese, 100% **Nationality**: NR	**Methods:** QIAamp RNA Blood Mini Kit and RNase-Free DNAse; Illumina Sentrix Human-6 v2 Expression Bead-Chips **Validation:** Q-RT-PCR for 4 genes found in the blood samples (*VAV3, TNF, PTPN6, CD48*); *ACTB* for internal control; strong agreement was observed in all genes Used FDR to adjust for multiple comparisons **Tissue:** Peripheral leukocytes	5197 genes in blood were differently expressed in GDM patients after adjustment for multiple comparisons. 52% ↑ GDM vs Control; 48% ↓ GDM vs Control. Genes relevant to GDM pathogenesis: *KLRK1* (NKG2D) *HCST* (DAP10) *IFNG* *TNF* *IL1B* *LEP* *HLA-G* *VAV3* *PTPN6* *CD48* *IL15* 32 enriched pathways; 8 pathways (25% of total) were related to immunity and inflammation. Others included cancer-related pathways, infectious diseases, cell growth and death, metabolic disorders, endocrine system and signal transduction. Top 6 enriched KEGG pathways: natural killer cell mediated cytotoxicity, epithelial cell signaling in Helicobacter pylori infection, apoptosis, toll-like receptor signaling pathway, proteasome, and antigen processing and presentation.

NR=not reported; GDM=gestational diabetes; T2D=type 2 diabetes; NK=natural killer cells; RNA=ribonucleic acid; DNA=deoxyribonucleic acid; PCR=polymerase chain reaction; RT-PCR=Reverse transcription polymerase chain reaction; Q-RT-PCR=quantitative reverse transcription polymerase chain reaction; mRNA=messenger ribonucleic acid; HgbA1c=hemoglobin A1c; RNA-Seq=ribonucleic acid sequencing; RPLP0= Ribosomal Protein Lateral Stalk Subunit P0 gene; ACTB=Actin Beta; HPRT1= hypoxanthine phosphoribosyltransferase 1; G6PD= Glucose-6-phosphate dehydrogenase; TKT=Transketolase; ALDOA= Aldolase, Fructose-Bisphosphate A; PGLS= 6-Phosphogluconolactonase; DCXR= Dicarbonyl And L-Xylulose Reductase; IGFBP=Insulin-Like Growth Factor Binding Protein; VAV3=Vav Guanine Nucleotide Exchange Factor 3; TNF=tumor necrosis factor; PTPN6= Protein Tyrosine Phosphatase Non-Receptor Type 6; CD48=CD48 Molecule; FDR=False Discovery Rate; KLRK1= killer cell lectin like receptor K1; NKG2D=Natural Killer Group 2D; HCST=Hematopoietic Cell Signal Transducer; DAP10= deoxyribonucleic acid polymerase III-activating protein of 10KDa; ITAM=Immunoreceptor Tyrosine-Based Activation Motif; INFG=Interferon Gamma; IL1B=Interleukin 1-Beta; LEP=Leptin; HLA-G=Human Leukocyte Antigen G; IL15=Interleukin 15; KEGG= Kyoto Encyclopedia of Genes and Genomes

*The validation genes and gene findings as reported in the abstract do not match the text of the manuscript; Findings from the body of the manuscript were reported because the narrative matches the table of results. See further discussion in risk of bias analysis and limitations sections. The up arrow means upregulated and the down arrow means downregulated.

**Table 2:** Summary of micro-RNA (miRNA) studies included in the final sample.

Author (Year)	Study Purpose, Design, Setting	Sample	Instrumentation/Methods	Key Findings
Cao et al. (2017)	**Purpose:** To determine if miR-16-5p, miR-17-5p, and miR-20a-5p can serve as diagnostic markers for GDM and what their relationship is to different GDM factors (BMI, insulin resistance, and tumor necrosis) **Design:** Observational **Setting:** Tianjin Central Hospital of Gynaecology Obstetrics **Location:** Tianjin, China	**Sample:** *N* = 157 n = 85 GDM n = 72 Control **Age (years)**: Range & Standard Deviation NR; 26.8 ± 3.5 (GDM) 26.4 ± 3.6 (Control) **BM**I: Range & Standard Deviation NR; 25.1 ± 2.8 (GDM) 23.4 ± 2.3 (Control) **Gestational Age at Sampling (weeks)**: Range 24-28; 25.8 ± 2.5 (GDM) 26.1 ± 1.2 (Control) **Race:** NR **Ethnicity:** NR **Nationality:** NR	**Methods:** 3 time points - q4 weeks until GDM diagnosis. RNeasy plus mini kit qRT-PCR was used to quantify miRNAs (miR-16-5p, miR-17-5p, miR-19a-3p, miR-19b-3p, miR-20a-5p) from plasma. **Validation**: NR **Tissue:** Plasma	↑: miR-16-5p, miR-17-5p, miR-20a-5p in pregnant people with GDM compared with controls at each time point. Positive correlation between these miRNAs and insulin resistance, but not with TNF-α or BMI.
Gillet et al. (2019)	**Purpose:** To determine if there is differential expression of 17 miRNAs in circulating extracellular vesicles (EVs) in GDM and Control pregnant women. **Design:** Observational **Setting:** Centre Hospitalier Universitaire **Location:** Sherbrooke, QC, Canada	**Sample:** *N* = 69 n = 23 GDM n = 46 Control **Age (years):** Range & Standard Deviation NR**;** 29.8 ± 5.3 (GDM) 27.9 ± 4.4 (Control) **BMI (kg/m^2):** Range & Standard Deviation NR; 28.2 ± 7.2 (GDM) 24.5 ± 4.7 (Control) **Gestational Age at Sampling (weeks):** Range 6-15 weeks; 10.5 ± 2.5 (GDM) 10.6 ± 2.4 (Control) **Race**: NR **Ethnicity**: NR **Nationality**: Canadian (92.8%); Other (7.2%)	**Methods:** Q-RT- PCR EV presence confirmed as early as 8 weeks gestation. 4335 initial targets narrowed to 58 targets involved in 257 pathways. 3 pathways selected for this analysis: the type 2 diabetes mellitus signaling pathway, the insulin receptor signaling pathway, and the AMP-activated protein kinase (AMPK) signaling pathway. **Validation**: Internal validation per Brosseau et al. **Tissue:** Serum extracellular vesicles	10 of the 17 miRNAs (miR-122-5p, miR-132-3p, miR-1323, miR-136-5p, miR-182-3p, miR-210-3p, miR-29a, miR-29b-3p, miR-342-3p, and miR-520h) were ↑ in GDM vs control. Enriched pathways: insulin receptor signaling pathway, AMPK signaling pathway, and epidermal growth factor receptor-phosphatidylinositol 3-kinase-Akt pathway–involved in placental development, fetal growth, and insulin and glucose regulation.
Hromadnikova et al. (2020)	**Purpose:** To conduct risk assessments for developing diabetes mellitus, cardiovascular and cerebrovascular diseases in patients who have had GDM 3-11 years post-delivery using epigenetic modifications of miRNA. **Design:** Observational **Setting:** NR **Location**: Czech Republic	**Sample:** *N* = 200 n = 111 GDM (93 GDM on diet; 18 GDM on diet & therapy) n = 89 Control **Age (years):** Range & Standard Deviation NR; 38.70 ± 0.37 (GDM on diet) 38.61 ± 0.80 (GDM on diet & therapy) 38.33 ± 0.38 (Control) **BMI (kg/m^2):** Range & Standard Deviation NR; 23.85 ± 0.37 (GDM on diet) 27.21± 0.83 (GDM on diet & therapy) 23.15 ± 0.38 (Control) **Gestational Age at Sampling (weeks):** Not Applicable (data collected 3-11 years post-delivery) **Race:** Caucasian, 100% **Ethnicity:** NR **Nationality:** NR	**Methods:** RT-PCR **Validation**: NR **Tissue:** Whole blood	The expression of 26 miRNAs (miR-1-3p, miR-16-5p, miR-17-5p, miR-20a-5p, miR-20b-5p, miR-21-5p, miR-23a-3p, miR-24-3p, miR-26a-5p, miR-29a-3p, miR-100-5p, miR-103a-3p, miR-125b-5p, miR-126-3p, miR-130b-3p, miR-133a-3p, miR-143-3p, miR-145-5p, miR-146a-5p, miR-181a-5p, miR-195-5p, miR-199a-5p, miR-221-3p, miR-342-3p, miR-499a-5p, and miR-574-3p) ↑ in women previously affected with GDM compared to controls, even on average 5 years postpartum. Combined screening of 16 of those miRNAs (miR-1-3p, miR-16-5p, miR-17-5p, miR-20b-5p, miR-21-5p, miR-23a-3p, miR-26a-5p, miR-29a-3p, miR-103a-3p, miR-133a-3p, miR-146a-5p, miR-181a-5p, miR-195-5p, miR-199a-5p, miR-221-3p, and miR-499a-5p) showed the highest accuracy to detect mothers with a prior exposure to GDM (AUC 0.900, p < 0.001, sensitivity 77.48%, specificity 93.26%, cut o_ >0.611270413). It was able to identify 77.48% of mothers with an ↑ cardiovascular risk at 10.0% false positive rate. No difference in miRNA expression profiles between GDM on diet only and GDM on the combination of diet + therapy Predicted miRNA targets reflect pathways related to insulin signaling, type 1 diabetes mellitus, and type 2 diabetes mellitus
Lamadrid-Romero et al. (2018)	**Purpose:** To determine if 12 neural development miRNAs are altered in GDM **Design:** Observational **Setting:** Instituto Nacional de Perinatología **Location:** Mexico City, Mexico	**Sample:** *N* = 151 n = 67 GDM 1^st^ Trimester: 27 2^nd^ Trimester: 26 3^rd^ Trimester: 21 n = 74 Control 1^st^ Trimester: 14 2^nd^ Trimester: 26 3^rd^ Trimester: 27 **Age (years)**: NR (Range 18 -35) **BMI (kg/m^2)**: NR (below 29 as inclusion criteria) **Gestational Age at Sampling (weeks)**: NR **Race**: NR **Ethnicity**: NR **Nationality**: NR	**Methods:** TRIzol reagent; qRT-PCR Different sample sizes were analyzed for different miRNAs. **Validation**: NR **Tissue:** Serum	↑ miR-183-5p, miR-200b-3p, miR-125-5p, and miR-1290 were detected during the first trimester for GDM group. ↑ miR-183-5p, miR-200b-3p, miR-125-5p and miR-137 were detected in the second and third trimester, respectively.
Pfeiffer et al. (2020)	**Purpose:** To determine if there is a signature in 4 circulating miRNAs of interest in lean women with GDM who have no insulin resistance risk factors **Design:** Observational **Setting:** Endocrinology and Pregnancy Clinic, Puerta del Mar University Hospital, Cadiz **Location:** Cadiz, Spain	**Sample:** *N* = 60 n = 31 GDM n = 29 Matched Control **Age (years):** Range & Standard Deviation NR; 31.9 ± 1.8 (GDM) 31.0 ± 3.6 (Control) **BMI (kg/m^2):** Range & Standard Deviation NR; 22.5 ± 1.8 (GDM) 22.3 ± 1.8 (Control) **Gestational Age at Sampling (weeks):** Range 26-30; NR (GDM) NR (Control) **Race**: NR **Ethnicity**: NR **Nationality**: NR	**Methods:** miRNeasy Serum/Plasma kit (Qiagen) Q-RT-PCR 4 miRNAs measured: miR-224, miR-103-3p, miR-206, and miR-330-3p Multivariable logistic regression model included age, pregestational BMI, weight gain, triglycerides, and the 4 miRNAs measured in this study. **Validation**: NR **Tissue:** Serum	miR-330-3p was 5.2-fold ↑ in the GDM group compared to control. ↑ levels of miR-330-3p in GDM group compared with control were associated with: 1. a ↑ proportion of spontaneous deliveries than cesarean section in GDM 2. ↑ levels in GDM with spontaneous deliveries 3. ↑ levels in GDM patients treated with diet, but not GDM treated with insulin 1031 gene targets of miR-330-3p predicted; insulin signaling pathway was one of 12 pathways overrepresented. miR-330-3p was significant independent predictor of GDM in the final model.
Pheiffer et al. (2018)	**Purpose:** To determine if serum miRNAs are regulated in South African women with GDM in a similar manner to other populations. **Design:** Observational **Setting**: Primary clinic **Location**: Johannesburg, South Africa	**Sample:** N = 81 n = 28 GDM n = 53 Control **Age (years)**: Range & Standard Deviation NR; 29.5 ± 6.2 (GDM) 28.6 ± 6.4 (Control) **BMI (kg/m^2):** Range & Standard Deviation NR; 28.1 (23.9-31.3) (GDM) 26.2 (21.9-29.8) (Control) **Gestational Age at Sampling (weeks)**: Range 24-28; 26.0 (24.0-28.0) (GDM) 27.0 (25.0-28) (Control) **Race:** Black, 100% **Ethnicity:** NR **Nationality:** South African	**Methods:** miRNeasy Serum/Plasma kit (Qiagen); MiScript miRNA PCR (miR-16-5p, miR-17-5p, miR-19a-3p, miR-19b-3p, miR-20a-5p, miR-29a-3p, miR-132-3p, and miR-222-3p) **Validation**: NR **Tissue:** Serum	miR-20a-5p, miR-222-3p significantly ↓ in South African women with GDM compared to healthy pregnant controls using an FDR cutoff of 0.15. miR-20a-5p and the presence of 1 or more risk factors was significant independent predictor for GDM Risk factors: advanced maternal age (age ≥ 35 years), obesity (body mass index ≥ 30 kg/m2), family history of diabetes mellitus, delivery of a previous baby >4 kg, glucosuria, recurrent pregnancy loss, stillbirth, or birth of a baby with congenital abnormalities. Age, BMI, and miR-222-3p were not significantly, independently associated with GDM. 53 KEGG pathways for regulation, various cancers, and insulin signaling were enriched by miR-20a-5p gene targets
Sorensen et al. (2021)	**Purpose**: To determine if a panel of 8 miRNAs at baseline could be used in the GDM prediction for obese pregnant women **Design:** Observation (nested case-control study) **Setting:** DALI Lifestyle Study of 9 European Countries **Location**: Austria	**Sample:** *N* = 123 n = 41 Control n = 41 early-GDM n = 41 late-GDM **Age (years):** Range & Standard Deviation NR; 33.2 ± 3.8 (Control) 33.7 ± 4 (early-GDM) 32.7 ± 4 (late-GDM) **BMI (kg/m^2)**: Range & Standard Deviation NR; 33.3 (32.2–35.4) (Control) 33.3 (31.7−36.0) (early-GDM) 33.3 (31.7−35.9) (late-GDM) **Gestational Age at Sampling (weeks):** Range <20 weeks (early-GDM); 24-28 (late-GDM); 15.2 ± 2.4 (Control) 14.9 ± 2.4 (early-GDM) 15.3 ± 2.5 (late-GDM) **Race**: NR **Ethnicity**: 90% European (Control) 80% European (early-GDM) 78% European (late-GDM) **Nationality**: NR	**Methods:** MultiScribe™ Reverse Transcription kit; RT-PCR PCR. **Validation**: NR **Tissue:** Serum	3 out of 8 miRNA (miR-16-5p, miR-29a-3p, and miR-134-5p) levels were ↑ at baseline in women who went on to develop GDM. Women with late-onset GDM had ↑ miR-16-5p and miR-122-5p than women with early-onset GDM. The 3 miRNAs combined differentiated between late GDM cases and healthy controls (AUC = 71.7%). Adding fasting plasma glucose increased AUC to 81.0%. Adding maternal heart rate, neck size, or maternal height individually to the combined 3 miRNAs increased AUC to 72.7%. 1890 unique targets identified; most linked to miR-29a-3p; 148 targets common between 2 or more miRNAs. Vascular endothelial growth factor (VEGF)-, fibroblast growth factor (FGF)-, phosphoinositide (PI)-3 kinase-, Notch- and insulin signaling pathways were found to be overrepresented of predicted targets of the miRNAs. miR-29a-3p, miR-134-5p and miR-16-5p positively correlated with 2-h fasting glucose levels measured at 24–28 weeks of gestation after adjustment for maternal age and BMI, gestational age, and offspring sex. miR-16-5p positively correlated with HgbA1C; miR-122-5p negatively correlated with insulin sensitivity, HDL cholesterol, and leptin; and miR-122-5p positively correlated with birthweight
Stirm et al. (2018)	**Purpose:** To determine if non-coding RNA in white blood cells play a role in patients with GDM compared to controls **Design:** Observational **Setting:** NR **Location:** Germany	**Sample:** Screening: *N* = 16 n = 8 GDM n = 8 Control Validation: *N* = 60 n = 30 GDM n = 30 NGT **Age (years):** Range & Standard Deviation NR; Screening 33 ± 5 (Control) 32 ± 3 (GDM) Validation 32 ± 4 (Control) 31 ± 4 (GDM) **BMI (kg/m^2):** Range & Standard Deviation NR; Screening 28.1 ± 5.4 (Control) 29.1 ± 6.1 (GDM) Validation 29.5 ± 5.6 (Control) 29.8± 4.07 (GDM) **Gestational Age at Sampling (weeks):** Range 24-32; Screening 23 ± 9.5 (Control) 25.9 ± 1.7 (GDM) Validation 27.6 ± 2.37 (Control) 27 ± 2.3 (GDM) **Race**: Caucasian, 100% **Ethnicity**: NR **Nationality**: NR	**Methods:** RT-q-PCR **Validation**: Internal and external validation methods used **Tissue:** Whole blood	29 miRNAs ↑ in GDM patients compared to controls miR-19a, miR-142, miR-143, miR-340, miR-7g, and miR-19b were selected for external q-PCR validation. miR-340 was found at ↑ levels in GDM *PAIP1*, a downstream target was significantly ↓ in GDM. The 4 miRNAs positively associated with BMI were unrelated to GDM. ↑ insulin or ↓ glucose reduced miR-340 expression.
Tagoma et al. (2018)	**Purpose:** To determine and compare the expression profiles of plasma miRNA in GDM and Control pregnant women. **Design:** Observational **Setting:** Tartu University Hospital Women’s Clinic **Location:** Estonia	**Sample**: *N* = 22 n = 13 GDM n = 9 Control **Age (years):** Range & Standard Deviation NR; 31.1 ± 4.2 (GDM) 28.1 ± 4.5 (Control) **BMI (kg/m^2):** Range & Standard Deviation NR; 28.4 ± 6.8 (GDM) 21.3 ± 1.7 (Control) **Gestation Age at Sampling (weeks)**: Range 23-31; 27.5 ± 1.9 (GDM) 25.3 ± 1.9 (Control) **Race:** NR **Ethnicity:** NR **Nationality:** NR	**Methods:** miRNeasy Serum/Plasma Kit (Qiagen); qRT-PCR using the miScript II RT Kit (Qiagen). **Validation**: Internal validation of 3 upregulated miRNAs using RT-PCR (miR-195-5p, miR-30d-5p, and miR-92a-3p) **Tissue:** Plasma	15 miRNAs were ↑ in GDM group, 41 significantly enriched pathways. The 15 miRNAs were miR-7e-5p, miR-7g-5p, miR-100-5p, miR-101-3p, miR-146a-5p, miR-18a-5p, miR-195-5p, miR-222-3p, miR-23b-3p, miR-30b-5p, miR-30c-5p, miR-30d-5p, miR-342-3p, miR-423-5p, and miR-92a-3p. miR-195-5p had highest fold upregulation out of top 3 validated miRNA (miR-195-5p, miR-30d-5p, and miR-92a-3p). Fatty acid biosynthesis and fatty acid metabolism pathways were overrepresented miR-195-5p targeted highest number of genes important in the fatty acid metabolism pathway.
Wander et al. (2017)	**Purpose:** To determine if levels of circulating candidate miRNAs during 7-23 weeks of pregnancy are related to GDM development **Design:** Observational **Setting:** Center for Perinatal Studies at Swedish Medical Center **Location:** Seattle, Washington, USA	**Sample:** *N* = 116 n = 36 GDM n = 80 Control **Age (years):** Range & Standard Deviation NR; 34.3 ± 3.6 (GDM) 32.9 ± 4.4 (Control) **BMI (kg/m^2):** Range & Standard Deviation NR; 25.5 ± 6.7 (GDM) 21.7 ± 4.1 (Control) **Gestational Age at Sampling (weeks):** Range 7.0-22.9; 15.1 ± 2.9 (GDM) 16.5 ± 2.3 (Control) **Race:** 67% White (GDM) 80% White (Control) **Ethnicity:** Hispanic ethnicity was included as a category of race, but details NR **Nationality:** NR	**Methods:** Exiqon miRCURY™ RNA Biofluids Isolation Kit; qRT-PCR for candidate miRNAs (miR-126-3p, miR-155-5p, miR-21-3p, miR-146b-5p, miR-210-3p, miR-222-3p, miR-223-3p, miR-517-5p, miR-518a-3p, and miR-29a-3p) **Validation**: NR **Tissue:** Plasma	After adjusting for gestational age at blood draw, ↑ miR-155-5p and miR-21-3p levels were associated with ↑ odds for GDM, while miR-146b-5p and miR-517-5p odds of GDM were borderline. After adjusting for gestational age at blood draw, maternal age, and pre-pregnancy BMI, only miR-21-3p remained significantly associated with ↑ odds of GDM. miR-21-3p and miR-210-3p were detected in GDM overweight/obese but not lean women. Six miRNAs (miR-155-5p, -21-3p, - 146b-5p, -223-3p, -517-5p, and -29a-3p) were detected in GDM patients carrying male fetuses.
Yoffe et al. (2019)	**Purpose:** To determine which miRNAs measured during the first trimester are best for early GDM detection and differentiation. **Design:** Observational **Setting:** Careggi University Hospital (Italy); August Pi i Sunyer Biomedical Research Institute (Spain) **Location:** Italy and Spain	**Sample:** Initial Cohort: *N* = 43 n = 23 GDM n = 20 Control Validation Cohort: *N* = 20 n = 10 GDM n = 10 Control **Age (years)**: Range & Standard Deviation NR; 34 (32.5-37.5) (GDM) 34.5 (32.0-37.2) (Control) **BMI (kg/m^2):** Range & Standard Deviation NR; 28.6 (20.4-31.1) (GDM) 22.2 (20.1-31) (Control) **Gestational Age at Sampling (weeks):** Range 9-11 weeks; 10 (10.0-10.6) (GDM) 10 (10.0-10.2) (Control) **Race**: NR **Ethnicity**: NR **Nationality**: NR	**Methods:** miRNeasy Serum/Plasma Kit of collected blood samples; Nanostring ncounter profiled 798 human miRNAs GDM detection – machine learning models with leave-one-out cross validation (LOOCV) procedure **Validation**: External validation of the two upregulated miRNAs in a separate cohort using RT-qPCR; Internal validation using RT-qPCR **Tissue:** Plasma	22 samples from the initial cohort (51%) were removed from downstream analysis after quantifying miRNAs and prior to evaluating differently expressed miRNAs. In the remaining samples, miR-223 and miR-23a were found to be ↑ in GDM patients. When both miRNAs are used with a logistic regression model, this resulted in an AUC score of 0.91.
Zhao et al. (2011)	**Purpose:** To determine if serum miRNA and GDM have any associations; To determine if serum miRNA profiles could predict GDM before blood glucose changes **Design:** Observational (retrospective nested case-control study with multiple sites) **Setting:** Hospital system **Location:** Changzhou, China; Wuxi, China	*N*=152 *N*=48 (discovery stage) n=24 GDM n=24 Control Internal Validation Stage (n=36 per group) External Validation Stage (n=16 per group) **Age (years)**: Range & Standard Deviation NR; 28.8 ± 2.2 (GDM) 29.5 ± 1.9 (Control) **BMI (kg/m^2)**: Range & Standard Deviation NR; 21.4 ± 1.7 (GDM) 21.9 ± 1.8 (Control) **Gestational Age at Sampling (weeks)**: Range 16-19; 17.4 ± 0.7 (GDM) 17.2 ± 0.8 (Control) **Race**: NR **Ethnicity**: NR **Nationality**: NR	**Methods:** Qiagen miRNeasy Mini kit; TLDA Chips; Q-RT-PCR **Validation**: Internal and external validation methods used **Tissue:** Serum	10 miRNAs selected out of 73 total. miR-132, miR-29a, and miR-22 were found to be significantly ↓ in GDM. The AUC for the three miRNAs combined was greater (66.9%) than for individual miRNAs. miR-29a is inferred to be a negative regulator of glucose serum as knockdown of miR-29a led to ↑ *PCK2* expression. ↑ *PCK2* could then lead to an increase in glucose levels.
Zhu et al. (2015)	**Purpose:** To profile plasma miRNA that are differentially expressed in GDM patients compared to controls **Design:** Observational **Setting:** Zhongda Hospital, Southeast University, Nanjing, China **Location:** Nanjing, China	**Sample:** *N* = 20 n = 10 GDM n = 10 Control **Age (years):** Range & Standard Deviation NR; 30.03 ± 3.56 (GDM) 26.67 ± 4.59 (Control) **BMI (kg/m^2):** Range & Standard Deviation NR; 23.94 ± 2.98 (GDM) 19.24 ± 1.07 (Control) **Gestational Age at Sampling:** **(weeks):** Range 16-19; 17.66 ± 0.85 (GDM) 18.17 ± 0.93 (Control) Race: NR Ethnicity: NR Nationality: NR	**Methods:** miRNeasy Serum/Plasma Kit; RNA-seq **Validation**: Internal qRT-PCR used to validate 5 of the differentially expressed miRNAs using the Applied Biosystems 7500 Real-Time PCR System. **Tissue:** Plasma	32 miRNAs were differentially expressed in GDM with 20 being ↓ and 12 being ↑. 5 of the ↑ miRNAs (hsa-miR-16-5p, hsa-miR-17-5p, hsa-miR-19a-3p, hsa-miR-19b-3p, hsa-miR-20a-5p) were validated. 18 enriched pathways identified: endocytosis, mitogen-activated protein kinase (MAPK) signaling, insulin signaling, mTOR signaling, type 2 diabetes, Wnt signaling, proteoglycans in cancer, and transforming growth factor-β (TGF-β) signaling. Pathways suggest associations to insulin resistance and abnormal pregnancies.

NR=not reported; GDM=gestational diabetes; T2D=type 2 diabetes; RNA=ribonucleic acid; PCR=polymerase chain reaction; RT-PCR=reverse transcription polymerase chain reaction; Q-RT-PCR=quantitative reverse transcription polymerase chain reaction; HgbA1c=hemoglobin A1c; RNA-Seq=ribonucleic acid sequencing; TNF=tumor necrosis factor; FDR=false discovery rate; KEGG= Kyoto Encyclopedia of Genes and Genomes; BMI=body mass index; EV=extracellular vesicle; AMPK=adenosine monophosphate-activated protein kinase; AUC=area under the curve; HDL=high-density lipoprotein; PAIP1= polyadenylate-binding protein-interacting protein 1; PCK2= phosphoenolpyruvate carboxykinase 2, mitochondrial; MAPK=mitogen-activated protein kinase. The up arrow means upregulated and the down arrow means downregulated.

**Table 3:** Tools utilized by included studies for mRNA and miRNA gene targets and pathway analyses.

First Author (Year)	TargetScan*	DIANA v. 3 miRPath	Tarbase 7.0	PANTHER*	DAVID*	None/Not Applicable	Other Tools	Other: Explained
Sorensen et al. (2021)	**X**			**X**				
Pfeiffer et al. (2020)	**X**			**X**			**X**	EnrichR; Interpro Domains 2019; Pfamdomains
Zhu et al. (2015)	**X**				**X**		**X**	PicTar; miRanda
Stirm et al. (2018)	**X**						**X**	miRlastic R approach
Steyn et al. (2019)				**X**	**X**			
Pheiffer et al. (2018)		**X**	**X**					
Lamadrid-Romero et al. (2018)		**X**	**X**					
Tagoma et al. (2018)		**X**	**X**					
Gillet et al. (2019)							**X**	Qiagen Ingenuity Pathway Analysis
Hromadnikova et al. (2020)							**X**	miRwalk 2.0
Yoffe et al. (2019)							**X**	Gene Ontology Enrichment Analysis Software Toolkit (GOEAST)
Zhao et al. (2011)							**X**	Pathway Express (part of Onto-Tools) Cytoscape Software
Cao et al. (2017)						**X**		
Pappa et al. (2013)						**X**		
Wander et al. (2017)						**X**		
Zhao (2) et al. (2011)						**X**		

*Various versions. The shading/X means that the referenced study used that tool.

**Table 4:** miRNAs described in two or more studies.

**miRNA**	**Studies in Which miRNA was Described (*n)***	**Upregulated** ***n* (%)**	**Downregulated** ***n* (%)**	**No Difference** ***n* (%)**
miR-16-5p	5	4 (80%)	–	1 (20%)
miR-17-5p	4	3 (75%)	–	1 (25%)
miR-195-5p	2	2 (100%)	–	–
miR-20a-5p	4	3 (75%)	1 (25%)	–
miR-210-3p*	2	2 (100%)	–	–
miR-222-3p	3	1 (33%)	1 (33%)	1 (33%)
miR-29a	6	3 (50%)	1 (17%)	2 (33%)**
miR-342-3p	3	3 (100%)	–	–

*Associated with GDM in obese but not lean pregnant people

**Found only in pregnant people carrying fetuses assigned the sex of male at birth

miRNA – microRNA

### 3.1 Sample characteristics

Data were extracted from the included studies about participants’ age, race, ethnicity, nationality, BMI, and gestational age at the time of blood sampling (**Tables 1**, **2**). Overall, participants with GDM were slightly older than participants in the healthy control groups (*t*(1256) = 4.36 years, *p*<0.0001). The weighted mean age of the GDM group was 31.9 ± 3.0 years. The weighted mean age of the control group was 31.1 ± 3.5 years.

In the transcriptome studies, the participants’ nationality was reported in two studies, ethnicity was reported in one study, and race was reported in one study. The sample of participants from the transcriptome studies was 75% Greek nationality (22), 14% South African nationality and Black race (23), and 11% Chinese ethnicity (24). The articles did not define how race, ethnicity, or nationality were measured (i.e., self-identified, reference criteria, or other method). Seven of the 13 miRNA studies, representing 48.4% of miRNA participants, did not report race, ethnicity, or nationality of their participants (25–31). Of the miRNA studies that did, two reported nationalities, described as 12% South African; 10% Canadian; and 1% Other. Four studies reported race (12% Black, 55% White, 28% Other). One study reported ethnicity as European (15%) or Non-European (3%). Wander et al. (2017) included Hispanic ethnicity as a category in their race demographic, but they did not report the findings (32).

The time point in which BMI was measured varied by study. Five studies measured pre-pregnancy BMI (23, 29, 30, 32, 33). Ten studies measured BMI at the time of the blood sampling (23–25, 27–29, 31, 33–35). Five studies also measured gestational weight gain (22, 23, 30, 32, 33). The BMI ranges of included participants also varied by study. Overall, the BMI of the GDM group was significantly higher than the control group (*t*(1256) = 11.26 kg/m^2^, *p*<0.0001). The weighted mean BMI of the GDM group was 26.2 ± 3.2 kg/m^2^ and the control group was 24.2 ± 3.1 kg/m^2^. The participants in two of the three transcriptome studies had BMIs in the overweight and obese ranges. In ten of the miRNA studies, the BMI of the GDM group was significantly higher than control groups at the time of the blood draw. Two of the miRNA studies differentiated results unique to the lean participants in their samples (30, 32).

**Figure 2:**
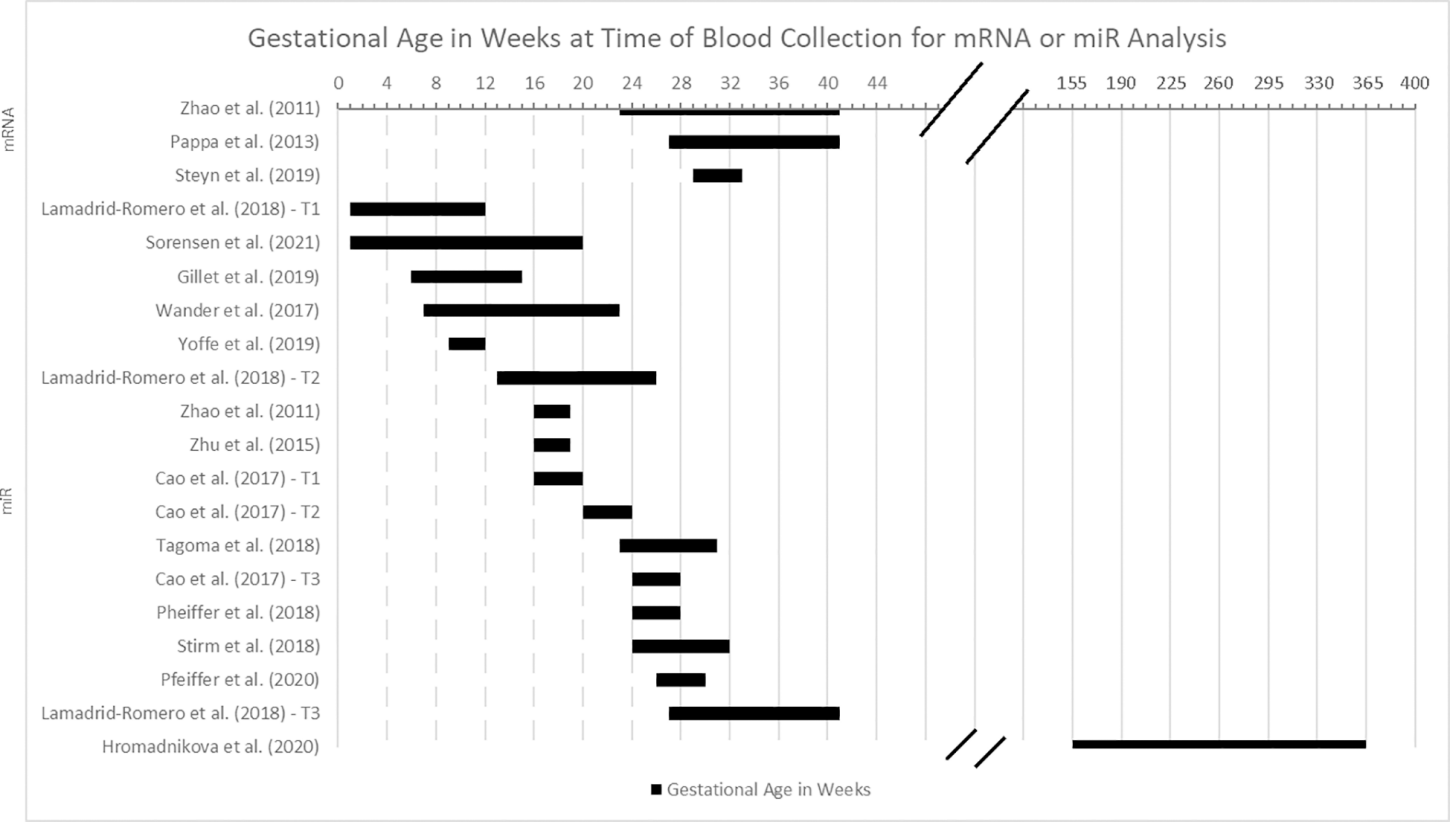
Gestational age in weeks at time of blood collection for miRNA or mRNA analysis.

**Figure 2** provides an overview of the gestational age at the time of the blood sample collection for mRNA and miRNA analysis. Although the gestational age at time of blood draw varied by study, there was no difference in the weighted mean gestational age of participants with GDM compared to controls for the overall sample.

### 3.2 Study characteristics

Data were extracted about the study design, GDM definition and diagnostic criteria used, exclusion criteria, tissue type, blood sample collection time points, and trimester represented for all included studies (**Tables 1**, **2**). All studies were observational and cross-sectional, except for two studies which measured the miRNAs at three time points each (25, 26).

The criteria used to define and diagnose GDM varied by study. Eight of the studies cited the International Association of Diabetes and Pregnancy Study Groups’ criteria (23, 25, 28, 29, 33, 35–37), six cited the ADA (22, 25–27, 31, 32), two cited the National Diabetes Data Group (24, 30), one cited Society of Obstetricians and Gynaecologists of Canada (34), and one referenced the World Health Organization (37). The exclusion criteria were also unstandardized. Self-reported measures or prior clinic records were used most often to exclude pre-gestational diabetes. Some studies excluded participants with specific conditions, fetal abnormalities, and/or complicated pregnancies from both groups (23, 25–27, 30, 31, 34–37) while another excluded complicated pregnancies only in the control group (32), and still others did not specify if there were exclusions except prior history of diabetes and carbohydrate metabolism disorders (22, 24, 28, 29, 33). Six of the studies included only singleton pregnancies (23, 27, 31, 32, 36, 37); the others did not specify whether the pregnancies reflected by the study results were singletons or multiple gestation.

The point in the pregnancy in which mRNAs and miRNAs were assessed was disparate across the studies, as detailed in **Figure 2**. In addition, the definition of a particular trimester by gestational age in weeks was unstandardized and inconsistently reported. For example, two studies measured only participants in their first trimester (29, 34), but they used different weeks to define the end of the first trimester (i.e., <12 weeks or <14 weeks). Four studies reported miRNA results discretely within the second trimester, which was defined as weeks 13-26 (25–27, 31), and three studies were discretely within the third trimester, defined as weeks 27 through the end of pregnancy (22, 23, 26). Two studies reported findings from the time period that corresponds with the standard of care for assessing for gestational diabetes (weeks 24-28), although this range spans late second trimester to early third trimester (25, 36). Others’ ranges spanned two or more trimesters (28, 31–33, 37).

### 3.3 Instrumentation, data acquisition, and data analysis

Instrumentation, data acquisition, and analysis methods were disparate across studies (**Tables 1**–**3**). The three transcriptome studies each used different methods for mRNA quantitation (22–24). One study used RNA-Sequencing methods (23), and another used microarray methods (24). Pappa et al. (2013) used real time quantitative polymerase chain reaction (qPCR) for quantitation since they evaluated a panel of ten “clock genes” associated with diurnal rhythms. Both Steyn et al. (2019) and Zhao et al. (2011) used reverse transcription qPCR (RT-qPCR) for internal validation (23, 24). Twelve out of the 13 miRNA studies (92%) used real time qPCR methods for miRNA quantitation (**Table 2**). The other study (8%) used the Nanostring ncounter platform (29). Internal and external validation methods were included in 54% and 23% of miRNA studies, respectively. **Table 3** provides an overview of the bioinformatics tools used in each of the studies where applicable.

### 3.4 Transcriptome studies

The transcriptome studies collectively identified 6,289 differentially expressed genes (DEGs). Overall, 2,702 DEGs (43.0%) were described as upregulated in participants with GDM compared to controls, while 2,499 DEGs (39.7%) were described as downregulated in GDM compared to controls. The candidate genes and target pathway findings are detailed in **Table 1**. The transcriptome pathways identified described various aspects of diabetes pathogenesis, including insulin and glucose signaling, regulation, and transport; natural killer cell mediated cytotoxicity; NADP and carbohydrate metabolism; immunity and inflammation; fatty acid biosynthesis and metabolism; and circadian clock rhythms.

The transcriptome studies analyzed correlations between mRNA levels and outcomes like glucose levels in the pregnant person, insulin resistance/insulin sensitivity, hemoglobin A1c (HbA1c), and birth weight. Glucose-6-phosphate dehydrogenase (*G6PD)*, insulin-like growth factor binding protein (*IGFBP-1)*, *IGFBP-2*, and transketolase (*TKT)* were inversely correlated with glucose levels in the pregnant person, measured at one or more time points. No significant correlation was found between *IGFBP-6* and glucose levels. Period circadian level 3 (*PER3)* was inversely correlated with HbA1c. *IGFBP-1* was positively correlated with the infants’ birthweight, while *IGFBP-2 and IGFBP-6* were not significantly correlated with birthweight.

### 3.5 miRNA studies

In the thirteen miRNA studies, 135 unique miRNAs were associated with GDM. Eight (miR-16-5p, miR-17-5p, miR-20a-5p, miR-29a-3p, miR-195-5p, miR-222-3p, miR-210-3p, and miR-342-3p) were described in two or more studies. See **Table 4** for details about the number of studies that described each miRNA, and whether or not it was found to be upregulated, downregulated, or no difference from healthy controls.

Within the studies, miRNA levels varied based on the time in pregnancy when GDM develops, the trimester or time period at which miRNAs were measured, sex of the fetus, obesity in the pregnant person, and treatment type (diet vs. pharmaceutical).

#### 3.5.1 Time in pregnancy

Two studies measured the miRNAs at multiple time points from the same participants (25, 26). In those studies, expression levels of miR-16-5p, miR-17-5p, and miR-20a-5p increased in each trimester as compared with the first trimester in the GDM group but remained constant in the control group (25). In contrast, miR-125b-5p increased over time from the first trimester to the third for the control group (26). Expression of miR-125b-5p was significantly higher in the GDM group compared to healthy controls in the first trimester, but then levels decreased 10-fold below those of the control group in the second trimester GDM samples (26). In control samples, expression levels of miR-183-5p, miR-200b-3p, and miR-125b-5p were the highest in the second trimester, whereas the highest level of miR-137 was observed in the third trimester (26).

When comparing first trimester GDM expression levels to controls, miR-183-5p, miR-200b-3p, miR-125b-5p, miR-1290 (26), miR-223, and miR-23a (29) were higher in the GDM group. During the second trimester, in the GDM group, the expression levels of miR-183-5p (26), miR-16-5p, miR-17-5p, miR-19a-3p, miR-19b-3p, miR-20a-5p (25, 27), were higher and miR-128-5p (26), miR-132, miR-29a, and miR-22 (31) were lower as compared with the control group. Finally, during the third trimester, in the GDM group, studies reported a higher level of miR-137 and lower levels of miR-183-5p and miR-200b-3p relative to the control group (26).

Four studies measured miRNA expression at the time of GDM diagnosis, between 24-28 weeks gestation and collectively identified 35 miRNAs that were significantly different in the GDM group compared to controls (25, 30, 33, 36). MiR-222-3p was significantly lower in South African pregnant people with GDM relative to controls (36). MiR-16-5p, miR-17-5p (25), miR-340 (33), and miR-330-3p (30) were significantly upregulated in the GDM group. Stirm et al. (2018) identified an additional 29 miRNAs that were significantly upregulated in the GDM group (false discovery rate <0.1) compared to controls, although miR-340 was the only one that was validated in their study (33). MiR-20a-5p was reported in two studies with mixed results. In one study conducted in China, miR-20a-5p was significantly higher in the GDM group, whereas in another study of South African pregnant people, miR-20a-5p was significantly lower.

One study measured miRNA levels in the formerly pregnant person an average of 5 years after birth of the baby (35). They found 26 miRNAs that were significantly higher in participants with a history of GDM compared to controls. A composite of 16 of the 26 miRNAs was identified as the best predictor of GDM exposure during pregnancy in their participants.

#### 3.5.2 Sex assigned at birth of the offspring

Two studies considered sex assigned at birth of the offspring in their analyses. Wander et al. (2017) differentiated results by sex of the offspring which was determined using retrospective chart review after delivery (32). They detected six miRNAs (miR-155-5p, miR-21-3p, miR-146b-5p, miR-223-3p, miR-517-5p, and miR-29a-3p) that were significantly different in the blood samples of participants with GDM carrying male fetuses. Conversely, Sorensen et al. (2021) found no sex-dependent differences in expression of miR-29a-3p and miR134-5p (37).

#### 3.5.3 Associations with BMI

In most studies, the GDM group had a significantly higher BMI at the time of the blood draw compared to the control group. Three studies reported findings for lean (i.e., pre-pregnancy BMI <25 kg/m^2^), overweight/obese (i.e., pre-pregnancy BMI ≥ 25 kg/m^2^), or obese (i.e., pre-pregnancy BMI ≥ 29 kg/m^2^) pregnant people (30, 32, 37). Sorensen et al. (2021) identified three miRNAs (miR-16-5p, miR-29a-3p, miR-134-5p) that were higher at baseline in obese women who went on to develop GDM (37). These three miRNAs combined differentiated those women who developed GDM earlier in pregnancy from those who developed GDM later in pregnancy, with the highest levels at baseline in women who developed GDM late in pregnancy. In the other two studies, miR-21-3p, miR-210-3p (32), and miR-330-3p (30) were significantly increased in obese but not lean women.

#### 3.5.4 Differences by treatment type

Two studies analyzed miRNA findings based on GDM treatment type (30, 35). Higher levels of miR-330-3p were associated with patients with GDM treated with diet, but not GDM treated with insulin, compared to controls (30). Hromadnikova et al. (2020) found no difference in miRNA expression profiles between GDM on diet only and GDM on the combination of diet plus pharmacologic therapy, however the pharmacologic group included 17 participants on insulin and 1 participant on metformin (35). Another study by Pheiffer et al. (2018) reported that their participants with GDM were treated with either metformin, insulin, and/or diet, but did not specify any details to characterize the sample or to differentiate results by treatment type (36).

#### 3.5.5 miRNA and health outcome correlations

Three studies described the relationships between miRNAs and related health outcomes (25, 33, 37). MiR-16-5p was positively correlated with insulin resistance (25), 2-hour fasting glucose levels at 24-28 weeks of gestation, and HbA1c (37). There was a positive correlation between miR-17-5p, and miR-20a-5p and insulin resistance, but not with tumor necrosis factor- α (TNF-α) or BMI (25). MiR-29a-3p and miR-134-5p were positively correlated with 2-h fasting glucose levels measured at 24–28 weeks of gestation after adjustment for maternal age and BMI, gestational age, and offspring sex (37). MiR-122-5p was negatively correlated with insulin sensitivity, high density lipoprotein (HDL) cholesterol, and leptin; and positively correlated with birthweight (37). Four miRNAs (miR-4473, miR-199-5a, miR-339-5p, and miR-3653-5p) were positively associated with BMI but were unrelated to GDM (33).

### 3.6 Risk of bias findings

Risk of bias was assessed in terms of recruitment and sampling methods, confidence in the assessment of exposure, confidence in assessment of the outcomes, matching between cases and controls, potential confounders, and missing data. Most studies described sample recruitment from eligible patients who were seen in a particular clinic. In most studies, the cases and controls were recruited during the same time. However, it is unknown to what extent these cases and controls were drawn from same population, and unknown how representative the samples are of the general population since sample characteristics were described inconsistently.

Confidence in assessment of exposure was assessed by considering the method of diagnosis of GDM and comorbidities. Variations in the GDM definition, diagnostic criteria referenced, and methods of GDM diagnosis were found across studies, although most were assessed between 24-28 weeks gestation. For example, the American Diabetes Association (ADA) recommends that pregnant people who test positive for diabetes during the first trimester be diagnosed with type 2 diabetes (T2D) (1). However, Lamadrid-Romero et al. (2018) classified those participants as having GDM because they had no prior risk factors for T2D (26). It is unknown to what extent undiagnosed T2D prior to pregnancy affected the results, due to the nature of the GDM diagnostic process.

Confidence in the individual studies’ assessment of the molecular markers was mixed. Most studies provided detailed descriptions of their analysis methods, but across the studies, instrumentation and analysis tools were unstandardized. One transcriptome study reported different results in the abstract versus the body of the manuscript. For the review of literature described in this paper, findings were reported from the body of the manuscript because the text in the results section matched the figures and discussion. Finally, validation of the mRNA and miRNA measurements was used only in a subset of studies and was not consistently applied in all studies.

Matching of exposed and unexposed participants for potential confounders affecting the outcome was done in a minority of studies. As expected, in most studies that measured fasting glucose and HbA1c, those levels were higher in the GDM group than the control group at the time of mRNA or miRNA analysis. BMI was also significantly higher in the GDM group in a majority of studies at the time of the blood draw. Other potential confounders such as pre-pregnancy BMI, weight gain during pregnancy, age of the pregnant person, gestational age at time of blood draw, singleton pregnancy, primiparous, behaviors during pregnancy such as smoking, and other sociocultural factors were recorded or adjusted for inconsistently across studies. It is also unknown if behavioral and environmental exposures were consistent among cases and controls because of limited sample characteristics reported. For example, it is unknown to what extent behaviors like physical activity, diet, smoking, exercise or psychosocial factors like stress, environmental factors, access to resources and prenatal care, health literacy, low socioeconomic status (SES), education, or structural racism resulting from being a member of racial ethnic minority groups, may be affecting results.

Missing data were described in some studies. For example, some studies analyzed different sample sizes for different time points (26), some had missing sample characteristics like age, BMI, or gestational age (24, 32), and some had missing blood samples and analyzed a smaller subset for the mRNA or miRNA results (32).

## 4 Discussion

This systematic review of the literature identified 16 articles that measured the transcriptome or miRNA expression in blood specimens of 684 adults with GDM compared to 691 healthy controls. Most studies applied an observational, cross-sectional design. Collectively, findings span the entire pregnancy from the first to the third trimester. Additional repeated measures studies are needed to compare mRNA or miRNA levels across gestational ages in the same participants.

The studies represent participants residing in 12 countries, with most set in China and South Africa. There is emerging evidence that differences in geographic location and ethnicity may be reflected in circulating miRNA levels (38, 39). Overall, most of the studies included in our review did not cite standardized definitions of race, ethnicity, and nationality to characterize the samples. To ensure high-quality precision healthcare that is equitable and representative, accurate sample descriptions are needed. Samples of diverse individuals should be recruited to ensure that the findings can be generalizable.

Overall, participants with GDM were slightly older with a significantly higher BMI at the time of the blood draw compared to controls. Few studies utilized either matching in their study design or controlled for confounders in their analyses. As age and weight may influence mRNA and miRNA levels, further research is needed to isolate the influence of GDM from other potentially confounding factors.

Three transcriptome studies were found that assessed circulating mRNA levels in pregnant people with GDM compared with healthy controls (22–24). All three studies represent mRNA levels in the third trimester. The transcriptome studies collectively identified 6,289 DEGs, of which 2,702 (43.0%) were described as upregulated in participants with GDM compared to controls, while 2,499 (39.7%) were described as downregulated in GDM compared to controls.

Expression levels of four mRNAs related to circadian clock rhythms were found to be significantly lower in pregnant people with GDM and were significantly correlated with HbA1c levels in one study (22). These four mRNAs were brain and muscle aryl hydrocarbon receptor nuclear translocator-like protein 1 (*BMAL1*), *PER3*, peroxisome proliferator activated receptor delta (*PPARD*), and cryptochrome circadian regulator 2 (*CRY2*). However, none of the ten circadian clock genes measured by Pappa et al. (2013) were reported in the supplementary results tables of the other two transcriptome studies (22–24). Differentially expressed clock genes are consistent with other studies that showed associations between sleep disruptions/night shift work and GDM (40). Specifically, sleep disruptions in GDM are associated with higher morning blood cortisol and glucose levels, increasing the need for long-acting insulin at night for patients with GDM. Previous studies about the role of circadian genes in T2D have indicated that *BMAL1* works in coordination with *CLOCK* as transcriptional activators of the circadian clock’s self-sustained transcriptional-translational feedback loops (41). These activators function as positive elements driving transcription of *PER*s, *CRY*s, and numerous other downstream elements involved in glucose metabolism and postprandial glycemia (41).

We extracted data from the transcriptome articles about the target pathways implicated by their findings and the tools they used to analyze them. Studies used different databases to determine the biological pathways related to their results. For example, Steyn et al. (2019) used the protein analysis through evolutionary relationships (PANTHER) and the database for annotation, visualization and integrated discovery (DAVID) tools (23), while Zhao et al. (2011) used Pathway Express (23, 24).

Collectively, the target pathways identified using these tools described various aspects of diabetes pathogenesis, including insulin and glucose signaling, regulation, and transport; natural killer cell mediated cytotoxicity; NADP and carbohydrate metabolism; immunity and inflammation; fatty acid biosynthesis and metabolism. The transcriptome pathways identified by the findings reflect the multi-system, complex nature of the disease (42). A prior study conducted by Flowers et al. (2022) assessed pathways targeted by differentially expressed miRNAs in people at risk for T2D and identified three themes (i.e., metabolism and inflammation, endocrine, and hormone) (43). Many of the implicated pathways identified by the studies included in this review also fit within these themes. Additional research is needed to validate transcriptome findings related to GDM in larger samples and additional settings and to determine how the pathophysiology of GDM may relate to the pathophysiology that underlies risk for T2D. Standardization is needed for data acquisition and analysis tools.

The miRNA study designs, participant characteristics, gestational age at the time of the miRNA blood analysis, and data analysis tools were disparate. Our analysis revealed that miRNA levels varied based on the time in pregnancy when GDM develops, the trimester or time period at which miRNAs were measured, sex of the fetus, obesity in the pregnant person, and treatment type (diet versus pharmaceutical). We delineated the miRNAs that were found to be significantly different in pregnant people with GDM by first trimester, second trimester, and the gestational age corresponding with GDM diagnosis (24-28 weeks gestation).

In the thirteen miRNA studies, 135 unique miRNAs were associated with GDM. Eight of these circulating miRNAs (miR-16-5p, miR-17-5p, miR-20a-5p, miR-29a-3p, miR-195-5p, miR-222-3p, miR-210-3p, and miR-342-3p) were the most validated for GDM. This list is updated from the previous review by Ibarra et al. (2018) by adding three miRNAs to the list (miR-195-5p, miR-210-3p, and miR-342-3p) and integrating any new findings about the five most-validated miRNAs previously identified (miR-16-5p, miR-17-5p, miR-20a-5p, miR-29a-3p, and miR-222-3p) (44). What is known from previous literature about each of these miRNAs in relation to GDM, other types of diabetes, obesity and weight change, or relevant pregnancy-related outcomes are discussed in more detail below. Animal model study findings are included in those cases where human study findings are unavailable.

### 4.1 miR-16-5p

MiR-16-5p is a powerful regulator of the insulin signaling pathway, pancreatic β-cell proliferation and apoptosis, and branched chain amino acids involved in insulin dysregulation (45–48). Target genes of miR-16-5p in mouse model and *in vitro* mechanistic studies include those that encode for insulin receptor substrate (IRS) proteins 1 and 2, the insulin receptor (INSR), and at least 24 other targets in the insulin signaling pathway like ak strain transforming (AKT) protein 1 and 3 (45–48). Other targets include genes encoding for the mammalian target of rapamycin (mTOR) protein and b-cell leukemia/lymphoma 2 protein (BCL-2) expression (45, 48), among others.

Our review found that miR-16-5p was upregulated compared to the control group in 80% (4 out of 5) of the included studies that reported it in their findings (**Table 4**). It was upregulated prior to onset of GDM in the first trimester (weeks 9-12) (48), during weeks 16-19 (27, 37), and in weeks 24-28 (25, 37, 48). Elevated gestational miR-16-5p may persist beyond pregnancy and may be permanently altered in women with GDM (35). However, in other studies conducted in Turkey, South Africa, and Poland, no significant difference was found in women with GDM compared with controls (36, 48–50). These mixed results may reflect regional environmental or cultural differences in miRNA expression levels, or instrumentation and study design differences, but further research is necessary to validate findings.

MiR-16-5p has been associated with traditional clinical indicators and long- and short-term outcomes. For example, elevated miR-16-5p is correlated with homeostatic model assessment of insulin resistance (HOMA-IR) (47, 48), insulin resistance (25), and cardiovascular disease risk (35), but not with preeclampsia (51). MiR-16-5p may also be associated with pre-pregnancy weight, because it has been reported to be elevated in overweight and obese women before week 20 gestation (37, 48). Further, it is consistently downregulated after surgical weight loss interventions in obese human and animal studies (46). Notably, in our sample of studies, miR-16-5p was not correlated with BMI in women with GDM (25). Future studies may seek to control for pre-pregnancy weight, weight category at the time of the blood draw, or gestational weight changes in their analyses for miR-16-5p to differentiate the effects of obesity from GDM pathology on miR-16-5p levels.

The findings from our review suggest that elevated baseline and first trimester miR-16-5p may be a predictor of late-onset GDM, particularly when combined with other miRNAs associated with GDM (37). These findings suggest that therapies that lower miR-16-5p prior to or early in pregnancy may help to prevent or decreased risk for GDM. However, more studies are needed to validate these findings, to understand regional or cultural differences, and to differentiate the contribution of obesity from pathology unique to GDM.

### 4.2 miR-17-5p

MiR-17-5p is involved in cell proliferation, inflammation, mitochondrial function, and diabetes-related vascular damage (48, 52). High glucose induction of human trophoblast cells *in vitro* led to upregulation of miR-17-5p in a simulated diabetic environment (52). There is some evidence that two enzymes associated with mitochondrial function, mitofusins 1 and 2, are targets of miR-17-5p (52).

In our sample, miR-17-5p was upregulated in 75% (3/4) of the studies (25, 27, 35). MiR-17-5p levels were upregulated compared with controls in weeks 16-19 (27) and 3-5 years post-pregnancy (35). During weeks 24-28 at the typical time of GDM diagnosis, expression levels were mixed. One study conducted in a sample of pregnant people in China found miR-17-5p levels to be upregulated (25), while two other studies of samples of South African and Turkish pregnant people, respectively, found no difference (36, 49). These mixed results may reflect cultural and environmental differences between the samples. Like miR-16-5p, miR-17-5p was positively correlated with insulin resistance but not TNF-α or BMI in one study (25). Also, like miR-16-5p, miR-17-5p has been linked to obesity, except that in the obese and coronary artery disease phenotypes the miR-17-5p levels were downregulated compared with healthy controls instead of upregulated as we found in our review (53). Further research is warranted to understand to what extent differences in miRNA expression levels are due to GDM pathology or obesity, and to describe how gestational miR-17-5p levels may change with weight changes.

### 4.3 miR-195-5p

In mouse and *in vitro* models, placental miR-195-5p targeted vascular endothelial growth factor A (*VEGFA*) in placental cells (54), and was an enhancer of zeste homolog 2 (*EZH2*) in umbilical cells (54), both of which may contribute to endothelial cell dysfunction and GDM progression. In the two studies that were included in our review, miR-195-5p was upregulated in pregnant people with GDM compared with controls in weeks 23-31 (28) and 3-5 years post pregnancy (35). This finding is consistent with previous work by Wang et al. (2020), who also found miR-195-5p to be upregulated at 25 weeks gestation in pregnant people with GDM compared with controls. MiR-195-5p targeted several of the genes important in the fatty acid metabolism pathway, including fatty acid desaturase 2 (*FADS2*), elongase of very long fatty acid 5, acetyl co-A carboxylase α *(ELOVL5)*, acetyl co-A synthetase 3 and 4 *(ACSL3, ACSL4)*, hydroxyacyl-CoA dehydrogenase trifunctional multienzyme complex subunit α *(HADHA)*, and carnitine palmitoyltransferase 1A genes *(CPT1A)* (28). This suggests that alterations in lipid metabolism, associated with changes in miR-195-5p expression, may be an important aspect of GDM pathogenesis and an area of focus for future study.

Mechanistic mouse model and *in vitro* studies about the role of miR-195-5p in GDM suggests that high levels of miR-195-5p inhibits cell proliferation and angiogenesis in human placental microvascular and umbilical endothelial cells treated with a high glucose condition (54, 55). The direction of the effect on cell apoptosis may vary by tissue, as high miR-195-5p inhibited apoptosis in umbilical endothelial cells (55) and increased apoptosis in the placental endothelial cells (54), both of which were undesirable effects contributing to cellular dysfunction. Additional studies are warranted to fully understand the mechanism of miR-195-5p in GDM pathogenesis. Two studies have measured associations between miR-195-5p and body mass and reported no association (35, 56), suggesting that obesity and gestational weight changes may not play a significant role in miR-195-5p levels in GDM. However, due to the connection between miR-195-5p and altered lipid metabolism, further validation of these results is warranted.

### 4.4 miR-20a-5p

The mechanism of miR-20a-5p’s involvement in GDM is unknown, but miR-20a-5p has been linked to cardiovascular disease, T2D, and pregnancy-related birth outcomes like small for gestational age and fetal growth restriction (57). In a study of participants undergoing coronary angiography, miR-20a-5p was associated with kidney function and estimated glomerular filtration rate after controlling for several confounders including T2D (58). In another study comparing participants with abdominal aortic aneurism with and without T2D, miR-20a-5p was associated with fructosamine concentration (59). MiR-20a-5p levels were significantly upregulated in the group with T2D compared with controls (59).

We found that miR-20a-5p was upregulated in pregnant people with GDM compared with controls in 75% of studies (3/4) in the final sample in this review (25, 27, 35). Levels were downregulated in the other 25% (36). For the studies that showed upregulated expression in pregnant people with GDM compared to controls, two of the studies were done in China (25, 27) and the other from the Czech Republic (35). Expression levels of miR-20a-5p were upregulated compared with controls across gestational periods and beyond—in weeks 16-19 (27), weeks 24-28 (25), and 3-7 years post-pregnancy (35). The average BMI for most study groups (pregnant people with GDM or control) in the three studies that reported upregulation had BMIs in the normal range. In contrast, in a study of South African pregnant people, miR-20a-5p was significantly lower in participants with GDM than participants in the control group in weeks 24-28 (36). The difference in findings may reflect regional or ethnic differences between the samples, but notably, the South African participants were more obese than participants in the other three studies. Previous mouse model studies have linked miR-20a-5p with induction of adipogenesis and lipogenesis *via* a novel regulatory circuit called CCAAT/enhancer-binding protein α/miR-20a-5p/Transducer Of ERBB2, 2 or TOB2 (60). Yet in the one study in our review that tested correlations between the miRNAs and clinical measures, miR-20a-3p was not correlated with BMI, but it was positively correlated with insulin resistance (25). Further research is warranted to validate previous findings, to understand the relationships between pre-gestational BMI, gestational weight gain or loss, and miR-20a-5p, and to distinguish the influence of obesity versus GDM pathology on miR-20a-5p expression levels.

### 4.5 miR-210-3p

MiR-210-3p is involved in hypoxia, insulin resistance, and anti-angiogenesis (34). Overexpression of miR-210-3p is predicted to inhibit insulin binding to the insulin receptor protein, the function of AMPK, and ultimately, β-oxidation and glucose transport (34). MiR-210-3p in late pregnancy has been positively associated with gestational age at birth (61). We found that results for miR-210-3p were mixed. Gillet et al. found it to be upregulated in pregnant people with GDM compared with controls (34), while Wander et al. found no difference (32). In one sample, miR-210-3p was associated with GDM in pregnant people with overweight or obesity but not those participants who were lean (32).

### 4.6 miR-222-3p

The role of miR-222-3p in GDM is unknown, but previous mechanistic studies implicate circulating miR-222-3p levels in both obesity and T2D (62–65). MiR-222-3p targets at least three experimentally validated genes of which low levels have been associated with T2D pathology: O-6-Methylguanine-DNA Methyltransferase (*MGMT*), Serine/threonine-protein phosphatase 2A 55 kDa regulatory subunit B-α isoform (*PPP2R2A*), and Reversion Inducing Cysteine Rich Protein With Kazal Motifs (*RECK*) (62, 63). In a mouse model mechanistic study, miR-222-3p also mediated the therapeutic effects pioglitazone, an oral hypoglycemic drug used for T2D, on skeletal muscle tissue, independent of the PPARγ mechanism-of-action of the drug (66).

In mouse models of obesity, overexpression of miR-222-3p was associated with increased adiposity by targeting DNA damage inducible transcript 4 (*Ddit4*) in adipocyte specific miR-221/222 knockout, which was associated with the suppression of the tuberous sclerosis complex 2 (TSC2)/mTOR complex 1 (mTORC1)/ribosomal protein S6 kinase (S6K) pathway (65). Circulating levels of miR-222-3p have been downregulated post-bariatric surgery induced weight loss in at least two studies (67). Paradoxically, however, circulating levels of miR-222-3p increased rather than decreased in overweight and obese human participants after a diet-induced weight loss intervention (68).

In our systematic review, results for miR-222-3p in pregnant people with GDM compared with controls were mixed, with one each downregulated during weeks 24-28 gestation (36), upregulated between weeks 23-21 gestation (28), and no difference during weeks 7-22.9 gestation (32). These three studies took place in different countries (South Africa, Estonia, and the United States, respectively). Although the difference was significant between the pregnant people with GDM and controls, miR-222-3p was not significantly, independently associated with GDM in a logistic regression model by Pheiffer et al. (36). While these three studies (28, 32, 36) did not describe correlations between miR-222-3p and any clinical parameters or outcomes, a validation study by Filardi et al. (2022) found that miR-222-3p was positively correlated with fasting blood glucose and birth weight in the third trimester in pregnant people with GDM (62). Overall, the highly disparate findings in studies about T2D, obesity, weight loss, and GDM suggest a complex mechanism that warrants further research.

### 4.7 miR-29a-3p

In our review, miR-29a-3p was the most validated because it was reported in 46% of the articles in our final sample, but results were mixed. MiR-29a-3p was upregulated compared with controls in 50% (3/5 studies) (34, 35, 37) and downregulated in 17% (1/5 studies) (31). Two studies (33%) found no difference (32, 36). Zhao et al. (2011) inferred miR-29a-3p to be a negative regulator of serum glucose because knockdown of miR-29a led to increased mitochondrial phosphoenolpyruvate carboxykinase 2 (*PCK2)* expression (31). Increased *PCK2* could then lead to an increase in glucose levels. However, their study was the only one in our sample in which the levels of miR-29a-3p were lower in pregnant people with GDM than controls, so the collective evidence does not yet support this inference. Further research is warranted to fully understand the mechanism and how it may vary by region or ethnicity.

### 4.8 miR-342-3p

The synthesized evidence from our review suggest that not only is miR-342-3p linked to complications of diabetes as previously proposed (18, 69), but it also may be an early indicator of risk. In the three studies in our review that described miR-342-3p levels, the miRNA was upregulated in 100% of them, spanning early to mid-pregnancy and several years post-pregnancy in diverse geographic locations (28, 34, 35). These three study samples were comparable in mean BMI by study group. In type 2 diabetes, miR-342-3p is predicted to inhibit *GLUT2* and trigger impaired insulin secretion in pancreatic β islet cells (34). It is one of the miRNAs that has been shown to be dysregulated across types of diabetes, including GDM, T1D, T2D (18, 70), and in people with obesity (71, 72). For people with metabolic syndrome at risk for diabetes, miR-324-3p is one of at least 49 miRNAs associated with insulin resistance in at least three studies (73). MiR-342-3p may also operate as a novel epigenetic integrator linking adipogenic homeostasis and angiogenesis (69). As such, it may be a useful early marker of metabolic risk. In mouse models of obesity, *SNAP25* was identified as a major target gene of miR-342-3p and the reduced expression of *SNAP25* may link to functional impairment hypothalamic neurons and excess of food intake (74). The inhibition of miR-342-3p may be a potential candidate for miRNA-based therapy for obesity (74). Due to miR-342-3p’s strong associations with obesity in animal studies, GDM studies should consider controlling for baseline obesity and gestational weight changes, or to consider differentiating from metabolically healthy people with obesity any potential mechanisms that are unique to GDM pathology.

### 4.9 Other subgroup analyses and overlap with T1D and T2D

Other subgroup analyses included differences in miRNAs by sex of the fetus or by GDM treatment type. Findings suggest that the sex of the fetus may affect maternal circulating miRNAs, however the two studies in our final sample that measured sex reported mixed results (32, 37). Further studies are warranted to better understand if or how sex of the offspring may influence GDM.

Only two studies in our final sample differentiated findings by GDM treatment type (diet versus diet with pharmacologic therapy) with mixed results. These results are complicated by mixing treatment types (i.e., insulin, metformin) within the group of participants with GDM, such as in Hromadnikova et al. (2020) included one participant on metformin with the 17 other participants who were treated with insulin (35). MiR-330-3p was the only miRNA found in our systematic review to vary by treatment group, with levels significantly higher in the GDM group treated with diet alone rather than diet plus insulin (30). Additional studies are needed to understand how miRNAs may vary within treatment groups, because metformin has been associated with the expression of miRNA levels in people undergoing treatment for insulin-resistant diseases (75). More research is needed to fully understand how metformin influences the expression of miRNAs in pregnant people with GDM.

The gene targets and miRNAs identified in this review mostly differ from those that have been previously emphasized for T1D and T2D, suggesting a distinct pathophysiology for GDM (76–78). Some exceptions are miR-16-5p (43), miR-29a-3p, miR-146a, miR-182, and miR-342-3p as previously mentioned in section 4.7 (18, 31–34, 36, 37, 43, 70, 76, 77, 79, 80). MiR-29a-3p was reported with mixed results in both GDM and T2D studies, with one or more study showing it to be upregulated and one or more showing it to be downregulated compared with controls. MiR-146a was significantly upregulated in both GDM and T2D studies. MiR-182 was reported to have significantly higher levels in GDM studies but was significantly downregulated in studies of T1D and T2D.

### 4.10 Risk of bias and limitations

The studies in the final sample are at moderate risk of bias due to several reasons, including the cross-sectional design, differences in sample characteristics between groups at the time of the analysis, inconsistencies in the standards used for GDM diagnosis, varying molecular quantitation methods and data analysis tools, and the handling of missing data. An international consensus is needed for GDM definitions and diagnostic criteria. More rigorous methods are needed to ensure that participants did not have pre-existing diabetes prior to pregnancy. We found that exclusion criteria were unstandardized, which adds additional complexity while synthesizing results across studies.

Replication and validation studies are needed before any of the mRNA or miRNA targets can be useful as clinical biomarkers or therapeutic targets. As this field of science continues to develop, efforts to standardize sample characterization, diagnostic guidelines, validation, and data acquisition and analysis tools and methods would strengthen the synthesis of results. Nonetheless, these studies represent the best available knowledge about transcriptional differences in the blood samples of adults with GDM compared to controls. This review was limited in that the search was conducted in only one database and only articles available in English were included in this analysis. Our ability to integrate the transcriptome studies with the miRNAome studies was limited by the findings available within each of the individual articles.

Many of the studies presented truncated findings or made available partial datasets. This limited our ability to search for overlapping gene targets and pathways between the miRNA and mRNA studies. Journals are increasingly requiring authors to make available full datasets with their publications. If applied consistently, this practice will allow for improved synthesis and validation of findings across multiple omics studies in the future.

### 4.11 Conclusions

Findings from this systematic review contribute new insights into the state of the science on transcriptomics and miRNA expression in blood from adults with GDM compared with healthy controls. Differences in expression of mRNA and miRNA levels were identified by gestational age at the time of the study, sex of the fetus, BMI of the pregnant person, and GDM treatment type. Metabolic pathways identified in these studies reflect the multi-system, complex pathophysiology of GDM. Eight miRNAs were found to be the most validated in the current literature: miR-16-5p, miR-17-5p, miR-20a-5p, miR-29a-3p, miR-195-5p, miR-222-3p, miR-210-3p, and miR-342-3p. With the exception of miR-222-3p, the most-validated miRs were upregulated in adults with GDM compared with controls in a majority of the studies that reported about them. The reasons for differences in the direction of change in the results (upregulated, downregulated, no difference) across studies are unknown, but may be related to confounding effects like maternal obesity, gestational weight changes, geographic or ethnic differences, instrumentation and data analysis differences, and study designs. Additional research, particularly with repeated measurement designs, is warranted to validate and refine the evidence, with an emphasis on standardization of research methods and recruiting diverse samples with sufficient power for subgroup analyses.

## Data availability statement

The original contributions presented in the study are included in the article/supplementary material. Further inquiries can be directed to the corresponding author.

## Author contributions

All authors reviewed and approved the final version of the manuscript. EF, KL, JC, BA, LJ-P, MM, BP, LR, and KR contributed to the conceptualization of the work and critically revised the manuscript. KL and JC completed the search, data extraction, and data analysis. KL was lead author. KL and LC wrote and formatted the data tables and figures.
